# Proteasome dysfunction induces muscle growth defects and protein aggregation

**DOI:** 10.1242/jcs.150961

**Published:** 2014-12-15

**Authors:** Yasuo Kitajima, Yoshitaka Tashiro, Naoki Suzuki, Hitoshi Warita, Masaaki Kato, Maki Tateyama, Risa Ando, Rumiko Izumi, Maya Yamazaki, Manabu Abe, Kenji Sakimura, Hidefumi Ito, Makoto Urushitani, Ryoichi Nagatomi, Ryosuke Takahashi, Masashi Aoki

**Affiliations:** 1 Department of Neurology, Tohoku University School of Medicine, 1-1 Seiryo-machi, Aoba-ku, Sendai 980-8574, Japan; 2 Department of Medicine and Science in Sports and Exercise, Tohoku University Graduate School of Medicine, 1-1 Seiryo-machi, Aoba-ku, Sendai 980-8574, Japan; 3 Department of Neurology, Kyoto University Graduate School of Medicine, Kyoto 606-8501, Japan; 4 Niigata University, Department of Cellular Neurobiology Brain Research Institute, Niigata 951-8510, Japan; 5 Department of Neurology, Wakayama Medical University Graduate School of Medicine, Wakayama 641-8510, Japan

**Keywords:** Proteasome, Autophagy, Skeletal muscle, Muscle atrophy

## Abstract

The ubiquitin–proteasome and autophagy–lysosome pathways are the two major routes of protein and organelle clearance. The role of the proteasome pathway in mammalian muscle has not been examined *in vivo*. In this study, we report that the muscle-specific deletion of a crucial proteasomal gene, *Rpt3* (also known as *Psmc4*), resulted in profound muscle growth defects and a decrease in force production in mice. Specifically, developing muscles in conditional *Rpt3*-knockout animals showed dysregulated proteasomal activity. The autophagy pathway was upregulated, but the process of autophagosome formation was impaired. A microscopic analysis revealed the accumulation of basophilic inclusions and disorganization of the sarcomeres in young adult mice. Our results suggest that appropriate proteasomal activity is important for muscle growth and for maintaining myofiber integrity in collaboration with autophagy pathways. The deletion of a component of the proteasome complex contributed to myofiber degeneration and weakness in muscle disorders that are characterized by the accumulation of abnormal inclusions.

## INTRODUCTION

The ubiquitin–proteasome and autophagy–lysosome pathways are the two major routes for protein and organelle clearance in cells (Braun and Gautel, 2011). These two systems are controlled by a transcriptional program that upregulates several crucial and rate-limiting enzymes (Glass, 2010; Jagoe and Goldberg, 2001). Proteasomal proteolysis is important in several organs; for example, proteasome inhibition using MG-132 leads to the cytoplasmic aggregation of TAR DNA-binding protein 43 (TDP-43) in cultured hippocampal and cortical neurons and in immortalized motor neurons (van Eersel et al., 2011). Similarly, the depletion of the 26S proteasome in mouse brain neurons causes neurodegeneration (Bedford et al., 2008).

The loss of skeletal muscle mass in humans at an older age, which is called sarcopenia, is a rapidly growing health issue worldwide (Vellas et al., 2013). The regulation of skeletal muscle mass largely depends on protein synthesis and degradation processes. Two muscle-specific E3 ubiquitin ligases, muscle RING finger 1 (MuRF1, also known as TRIM63) and muscle atrophy F-Box (MAFbx, also known as atrogin-1 or FBXO32), are thought to be key regulators of proteasomal proteolysis in skeletal muscle, especially under atrophy-inducing conditions (Cai et al., 2004; Sandri et al., 2004; Stitt et al., 2004). These proteins are markers of muscle atrophy because they are expressed at relatively low levels in resting muscle but are upregulated under a variety of atrophy-inducing conditions. The dysregulation of the proteasome system is also involved in several muscle diseases. Members of the ubiquitin-proteasome system are upregulated and the global ubiquitylation of proteins is increased in the muscles of dystrophic patients with laminin α2 chain deficiency. Interestingly, proteasome inhibition using MG-132 significantly improved the dystrophic phenotype (Carmignac et al., 2011). MG-132 also improves the dystrophic phenotype in a model of dystrophin deficiency (Bonuccelli et al., 2003; Winder et al., 2011). Therefore, the upregulation of proteasomal proteolysis likely leads to a reduction in skeletal muscle mass, which is in contrast to animal models of proteasomal dysfunction or downregulation in brain neurons that leads to degeneration. We hypothesized that the findings using MG-132 might involve proteasomal inhibition in non-muscle cells in the tissue. Because muscular dystrophy is characterized by inflammation, the effect of MG-132 on inflammatory cells must be considered.

Autophagy is another important cellular pathway for protein and organelle degradation. The efficiency of autophagic degradation declines during aging, leading to the accumulation of intracellular waste products (Salminen and Kaarniranta, 2009). Autophagy is an evolutionarily conserved degradative pathway through which long-lived intracellular proteins and organelles are delivered to the lysosome for destruction. This pathway is involved in the cellular response to starvation, cellular differentiation, cellular death, aging, cancer and neurodegenerative disease (Todde et al., 2009). The excessive activation of autophagy aggravates muscle wasting (Zhao et al., 2007). Interestingly, a study using mice with a muscle-specific deletion of *Atg7* revealed the upregulation of MuRF1 and atrogin-1, suggesting crosstalk between the autophagy and proteasomal pathways in skeletal muscle (Masiero et al., 2009). Proteasomal inhibition generally induces autophagy (Ding et al., 2007). Therefore, the role of the proteasomal pathway in skeletal muscle homeostasis should be evaluated while also considering autophagy and protein synthesis activity; however, the effect of proteasomal downregulation on autophagy using a loss-of-function strategy has not yet been described.

Proteasomal degradation is mediated by an ATP-dependent protease complex, the 26S proteasome, which is present in both the cytoplasm and nucleus. The 26S proteasome consists of a proteolytic, cylinder-shaped particle (the 20S proteasome) and an ATPase-containing complex (the 19S cap complex). The 19S cap complex unfolds ubiquitin-conjugated proteins to allow their entry into the 20S cylindrical particle. The 19S complex contains several putative ATPases, such as PSMC1–PSMC6. These subunits form a large family with a highly conserved ATPase domain (Sakao et al., 2000). PSMC4, also known as Rpt3, is an essential subunit of the 26S proteasome and is required for the degradation of most proteasomal substrates. In particular, Rpt3-deficient mice die before implantation owing to a defect in blastocyst development (Sakao et al., 2000). Interestingly, an insertion/deletion variant in intron 5 of the *Rpt3* gene was frequently found in a cohort of patients with Parkinson's disease (Marx et al., 2007). The combined knockdown of both *Rpt3 *and *Rpt6* caused defects in the assembly of regulated particles of the proteasome and led to diminished peptidase activity in HEK293T cells (Kaneko et al., 2009). Recently, we reported that the conditional knockout of the proteasome subunit *Rpt3* in motor neurons caused locomotor dysfunction that was accompanied by progressive motor neuron loss and gliosis in mice (Tashiro et al., 2012). Thus, the specific deletion of Rpt3 in skeletal muscle tissue might provide a better understanding of the role of the proteasome in muscle homeostasis without affecting other cell types in the tissue.

The working hypothesis of this study was that the downregulation of the ubiquitin proteasomal pathway might attenuate myocellular catabolic pathways to favor the maintenance of skeletal muscle mass. Thus, we generated conditional *Rpt3*-knockout mice to specifically block proteasomal activity in skeletal muscle to clarify the role of the proteasomal system in skeletal muscle tissue. Additionally, because the dysregulation of autophagy is involved in the pathogenic mechanisms of several myopathies, such as Pompe disease (Raben et al., 2008), Danon disease (Nishino et al., 2000), VMA21 deficiency (Ramachandran et al., 2013), autosomal dominant inclusion body myopathy associated with Paget's disease of the bone and frontotemporal dementia with valosin-containing protein (VCP) mutation (Watts et al., 2004), GNE myopathy (Li et al., 2013) and collagen VI muscular dystrophy (Grumati et al., 2010), we also investigated morphologically similar anomalies using specific immunohistochemical markers of known myopathies in the conditional *Rpt3*-knockout mice.

## RESULTS

### Generation of muscle-specific Rpt3-knockout mice

Rpt3-flox/flox mice were generated at Kyoto University, as described previously (Tashiro et al., 2012). Floxed Rpt3 mice (Rpt3-f/f) were crossed with a transgenic line expressing Cre recombinase under the control of a skeletal-muscle actin (ACTA1) promoter (Miniou et al., 1999) to generate muscle-specific Rpt3-knockout mice. However, an ACTA1-Cre-positive Rpt3-f/f mouse was not successfully generated when genotyping was performed in the F2 generation at 4 weeks of age. The examination of E18.5 embryo genotypes revealed ACTA1-Cre/Rpt3-f/f embryos, suggesting that this genotype causes embryonic lethality.

**Fig. 1.**
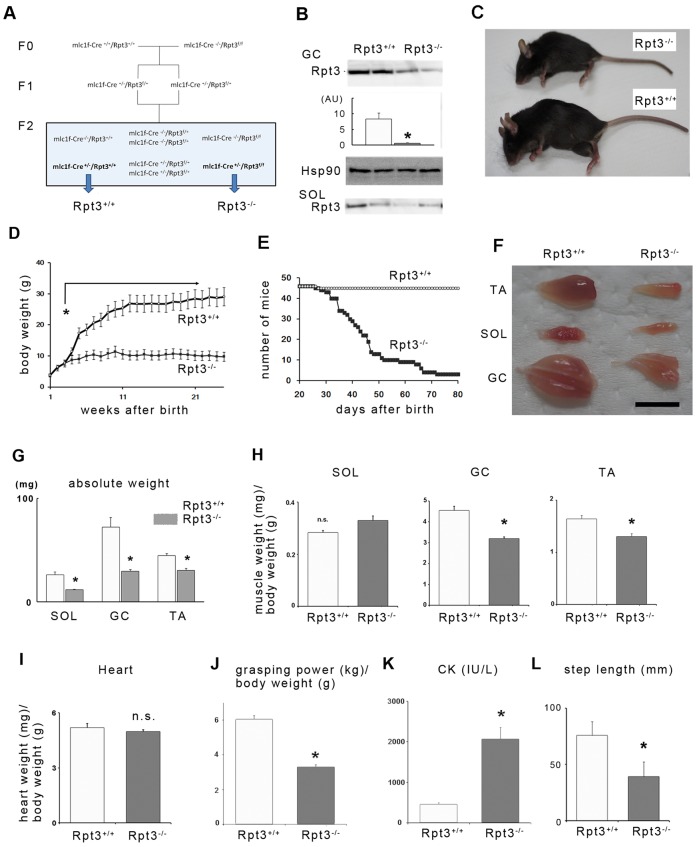
**Phenotypes of muscle-specific Rpt3-knockout mice.** (A) Generation of the Rpt3^−/−^ mice. Mlc1f-Cre^+/−^/Rpt3^f/−^ mice were mated to produce mlc1f-Cre^+/−^/Rpt3^+/+^ mice and mlc1f-Cre^+/−^/Rpt3^f/f^ mice (referred to as Rpt3^+/+^ mice and Rpt3^−/−^ mice, respectively). (B) Muscle homogenates were immunoblotted with antibodies against Rpt3 and Hsp90. Rpt3 protein was nearly undetectable in the homogenate of gastrocnemius (GC) from Rpt3^−/−^ mice. The homogenate from soleus muscle gave a similar result. Quantitative data are also presented (*n* = 3). White bar, Rpt3^+/+^ mice; gray bar, Rpt3^−/−^ mice. SOL, soleus; AU, arbitrary units. (C) The general appearance of Rpt3^−/−^ mice is shown. Note the smaller body frame and kyphosis in Rpt3^−/−^ mice. (D) The body weights of Rpt3^+/+^ and Rpt3^−/−^ mice are shown (*n* = 10 and 11, respectively). Note that the difference in the body weight became prominent after 3 weeks of age. Open circles, Rpt3^+/+^; closed squares, Rpt3^−/−^. (E) Kaplan–Meier survival curves for Rpt3^+/+^ and Rpt3^−/−^ mice (*n* = 46 each). (F) Appearance of excised skeletal muscles of Rpt3^+/+^ and Rpt3^−/−^ mice. TA, tibialis anterior. Scale bar: 1 cm. (G) Absolute skeletal muscle weight (mg) was significantly different between Rpt3^+/+^ and Rpt3^−/−^ mice in tibialis anterior, gastrocnemius and soleus muscles (*n* = 10). White bars, Rpt3^+/+^; gray bars, Rpt3^−/−^. (H) Skeletal muscle weight (mg)/body weight (g) were significantly different between Rpt3^+/+^ and Rpt3^−/−^ mice in tibialis anterior and gastrocnemius muscles (mostly fast-twitch fibers) but not in soleus muscle (half of the fibers are slow-twitch) (*n* = 10). (I) Heart weight (mg)/body weight (g) was not significantly altered in the Rpt3^−/−^ mice. (J) Grasping power (kg)/body weight (g) was significantly lower in the Rpt3^−/−^ mice (*n* = 5). (K) Creatine kinase (CK; IU/l) was significantly higher in the Rpt3^−/−^ mice (*n* = 5). (L) The step length (mm) was significantly shorter in Rpt3^−/−^ mice compared with Rpt3^+/+^ mice (*n* = 5). Quantitative data show the mean+s.e.m.; *P<0.05; n.s., non-significant [Student's *t*-test (B,G–I,K,L); Mann–Whitney U test (J)].

Rpt3-f/f mice were then crossed with a transgenic line expressing Cre recombinase under the control of a myosin light chain 1 fast (Mlc1f, also known as Myl1) promoter to generate muscle-specific Rpt3-knockout mice, hereafter referred to as Rpt3^−/−^ mice (Fig. 1A). Mlc1f-Cre/Rpt3^+/+^ mice were used as controls and are herein referred to as Rpt3^+/+^ mice (Fig. 1A). In Mlc1f-Cre mice, Cre activity was detected in skeletal muscle tissue, including the gastrocnemius, tibialis anterior and soleus muscles, but not in the heart (Bothe et al., 2000). *Mlc* transcripts are initially detected between E8.5 and E9.5 and are expressed robustly beginning at E10.5 (Mourkioti et al., 2008). Mlc1f expression is restricted to fast-twitch fibers in adults (Lyons et al., 1990), in contrast to the ACTA1 promoter, which becomes active in both the skeletal muscle and heart beginning at E9.5 (Miniou et al., 1999). Accordingly, Rpt3 protein was only slightly detectable in the gastrocnemius muscles, in which fast-twitch fibers predominate, of homozygous mice (Fig. 1B). Rpt3 protein was also markedly decreased in the soleus muscle (Fig. 1B). Mlc1f-promoter-driven Cre has an excision efficiency of 40–50% according to Southern blot analysis (Bothe et al., 2000). The trace amounts of persistent Rpt3 protein expression might therefore reflect non-excised floxed Rpt3. However, the presence of slow-twitch muscle fibers, endothelial cells, fibroblasts, macrophages, blood cells and mesenchymal cells might also contribute to the remaining expression. An immunoblotting analysis demonstrated multiple ladder bands bound by anti-Rpt3 (data not shown). The specificity of the Rpt3 antibody used was not high enough to obtain an Rpt3-specific immunohistochemical image.

### Proteasomal inhibition induces muscle growth defects and the loss of force production

The appearance of the resultant Rpt3^−/−^ mice was distinct from that of age-matched control Rpt3^+/+^ mice (Fig. 1C); Rpt3^−/−^ mice exhibited kyphosis and a smaller body frame. The growth curve showed a severe reduction in body growth, which differed from that of controls beginning at 3 weeks of age (Fig. 1D), whereas the survival curve suggested that the Rpt3^−/−^ mice had a reduced lifespan (Fig. 1E). Skeletal muscles also appeared smaller in the Rpt3^−/−^ mice (Fig. 1F); the absolute weights of the tibialis anterior, gastrocnemius and soleus muscles were smaller in Rpt3^−/−^ mice at 4 weeks of age (Fig. 1G). However, when muscle weight was evaluated per body weight, virtually no difference was detected between Rpt3^+/+^ and Rpt3^−/−^ animals in the soleus muscle, which is >50% slow-twitch fibers, whereas larger differences in fast-twitch-dominant muscles were observed between the animals (Fig. 1H). Additionally, the average heart weight was similar in both Rpt3^−/−^ and Rpt3^+/+^ mice (Fig. 1I), most likely because the Mlc1f promoter is not active in the heart.

The grasping strength of Rpt3^−/−^ mice was significantly lower than that of Rpt3^+/+^ mice, most likely because of the decreased muscle mass (Fig. 1J). Furthermore, creatine kinase levels were increased in Rpt3^−/−^ mice, suggesting the presence of muscle damage in the mutant mice (Fig. 1K). Rpt3^−/−^ mice also demonstrated a waddling gait, and their step lengths were markedly shorter compared with those of Rpt3^+/+^ mice (Fig. 1L).

### Morphological features of skeletal muscle in Rpt3^−/−^ mice

**Fig. 2.**
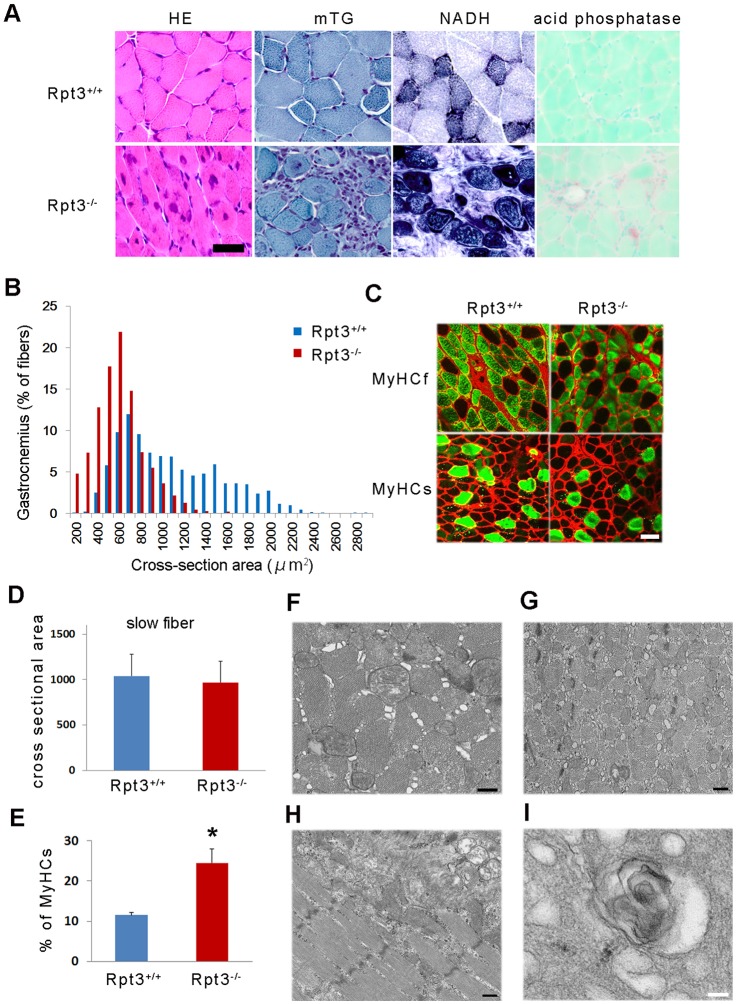
**Morphological changes in the muscles of Rpt3^−/−^ mice.** (A) Hematoxylin and eosin (HE) staining shows a general decrease in myofiber size along with the presence of central nuclei, basophilic inclusions and vacuolated fibers in the gastrocnemius of Rpt3^−/−^ mice at 4 weeks of age. Modified trichrome Gomori (mTG) staining revealed several basophilic inclusions in the myofibers and monocellular infiltrations in the interstitial perimysial space in Rpt3^−/−^ mice. NADH staining suggested that the myofibrils were disorganized in Rpt3^−/−^ mice. Acid phosphatase staining revealed mononuclear cell infiltrations around muscle fibers in Rpt3^−/−^ mice. Scale bar: 50 µm. (B) Quantification of the cross-sectional area of the myofibers in the gastrocnemius of Rpt3^−/−^ mice and Rpt3^+/+^ mice (*n* = 5). *P*<0.05 (Student's *t*-test). (C) Immunohistochemistry using myosin heavy chain slow (MyHCs) and fast (MyHCf) in the gastrocnemius of Rpt3^−/−^ and Rpt3^+/+^ mice. The diameter of the MyHCf-positive fibers in Rpt3^−/−^ mice was smaller; however, the same was not observed for the MyHCs-positive fibers. Red, laminin; green, MyHCf or MyHCs. Scale bar: 50 µm. (D) Quantitative data are also shown from the gastrocnemius of Rpt3^−/−^ and Rpt3^+/+^ mice (*n* = 200 for each fiber type). (E) The percentage of slow-twitch fibers was increased in the gastrocnemius of Rpt3^−/−^ and Rpt3^+/+^ mice (*n* = 5). Quantitative data show the mean+s.e.m.; *P<0.05 (Student's *t*-test). (F–I) Electron microscopy findings in the tibialis anterior muscles. Axial (F,G) and longitudinal (H,I) sections from 6-week-old Rpt3^+/+^ mice (F) and Rpt3^−/−^ mice (G–I). The myofibrils were smaller and the interstitial space was wider in the Rpt3^−/−^ mice. Sarcoplasmic reticulum dilation, filamentous structures and vacuolated structures were observed (H,I). Scale bars: 500 nm.

The examination of the skeletal muscle morphology in Rpt3^−/−^ mice revealed degenerative changes, the accumulation of basophilic inclusions in muscle fibers and centrally nucleated myofibers at 4 weeks of age (Fig. 2A). Mononuclear cell infiltration around muscle fibers was also observed (Fig. 2A). The myofiber cross-sectional area was decreased in Rpt3^−/−^ mice (Fig. 2B), indicating muscle fiber atrophy. As expected, fast-twitch muscle fibers were severely atrophied, whereas the average diameters of the slow-twitch fibers were approximately the same in both Rpt3^+/+^ and Rpt3^−/−^ mice (Fig. 2C,D). These findings indicate that the phenotypic change results from the deletion of the proteasomal component Rpt3 specifically in fast-twitch muscle fibers. In addition, the proportion of slow-twitch fibers was greater in the gastrocnemius muscle of Rpt3^−/−^ mice, which is most likely due to the degeneration of Rpt3-deficient fast-twitch fibers (Fig. 2E).

Next, the muscle tissue from the fast-twitch-dominant tibialis anterior muscle of Rpt3^−/−^ mice was examined by electron microscopy. The myofibrils in Rpt3^−/−^ mice were smaller in diameter than those in Rpt3^+/+^ mice (Fig. 2F,G). The distension of the sarcoplasmic reticulum and enlarged interstitial spaces were observed in Rpt3^−/−^ skeletal muscle tissue (Fig. 2F,G). In a subset of the muscle fibers, the distension of the sarcoplasmic reticulum (Fig. 2H,I) as well as vesicle formation and ruptured membranes were observed (Fig. 2I). In addition, a comparison of the electron micrographs obtained from the tibialis anterior muscles of Rpt3^−/−^ and Rpt3^+/+^ mice did not demonstrate increases in the number of autophagosomes and autolysosomes.

**Fig. 3.**
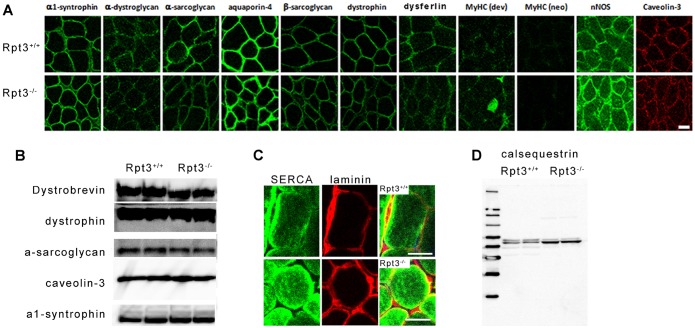
**Immunohistochemistry and immunoblotting of the dystrophin glycoprotein complex and sarcoplasmic reticulum proteins in the gastrocnemius of Rpt3^−/−^ and Rpt3^+/+^ mice.** (A,B) Immunohistochemistry and immunoblotting of the components of the dystrophin glycoprotein complex in 6-week-old mice. No apparent differences were observed in α1-syntrophin, α-dystroglycan, α-sarcoglycan, aquaporin-4, β-sarcoglycan, dystrophin, dysferlin, neuronal nitric oxide synthetase or caveolin-3 levels. A small amount of developmental myosin heavy chain was observed in muscles from Rpt3^−/−^ mice. Neonatal myosin heavy chain was not observed. Scale bar: 50 µm. (C,D) Immunohistochemistry and immunoblotting to detect the sarcoplasmic-reticulum-related proteins SERCA1 (C) and calsequestrin (D).

Furthermore, no marked defect or reduction in dystrophin or the components of the dystrophin–glycoprotein complex was observed (Fig. 3A,B). No apparent differences were observed in α1-syntrophin, α-dystroglycan, α-sarcoglycan, aquaporin-4, β-sarcoglycan, dystrophin, dysferlin, neuronal nitric oxide synthetase or caveolin-3 levels based on immunohistochemical examination (Fig. 3A). A small amount of developmental myosin heavy chain was observed in the gastrocnemius muscle tissue of Rpt3^−/−^ mice (Fig. 3A), suggesting the presence of a regenerative process that most likely counteracts fast-twitch muscle fiber degeneration, which leads to an increased serum creatine kinase level (Fig. 1K). The fluorescence corresponding to SERCA protein expression was higher in Rpt3^−/−^ gastrocnemius muscle tissue compared with that of Rpt3^+/+^ muscle tissue by immunohistochemical analysis (Fig. 3C). A higher level of calsequestrin in Rpt3^−/−^ gastrocnemius muscle was demonstrated by immunoblotting analysis (Fig. 3D). The increase in these sarcoplasmic reticulum proteins corresponds to the morphological change in the sarcoplasmic reticulum (Fig. 2F–I).

### Altered proteolysis in Rpt3^−/−^ mice

**Fig. 4.**
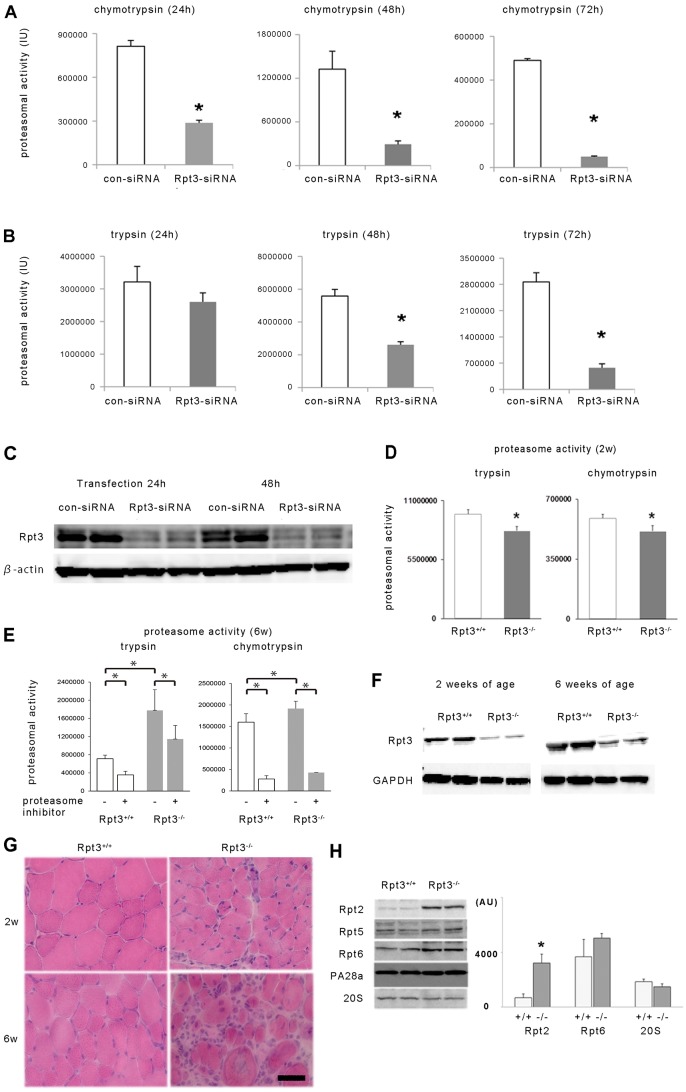
**The effect of Rpt3 knockdown and Rpt3 deletion on proteasomal activity *in vitro* and *in vivo*.** The chymotrypsin-like (A) and trypsin-like (B) activities of control (con-siRNA)- and Rpt3-siRNA-transfected C2C12 cells were assessed. Proteasomal activity was significantly suppressed in Rpt3-siRNA-transfected cells. Proteasomal activity at 24, 48 and 72 h after transfection is shown. IU, international units. (C) Rpt3 protein expression was suppressed in Rpt3-siRNA-transfected C2C12 cells. (D) Proteasomal activity (trypsin-like and chymotrypsin-like activity) in the tibialis anterior muscles of 2-week-old Rpt3^−/−^ and Rpt3^+/+^ mice (*n* = 5). (E) Proteasomal activity (trypsin-like and chymotrypsin-like activity) in the tibialis anterior muscles of 6-week-old Rpt3^−/−^ and Rpt3^+/+^ mice (*n* = 6). Trypsin-like and chymotrypsin-like activity were increased in Rpt3^−/−^ mice compared with Rpt3^+/+^ mice. The highly specific proteasomal inhibitor AdaAhx_3_L_3_VS (30 µM) significantly suppressed chymotrypsin-like activity by >70%, and trypsin-like activity by >30% from tibialis anterior muscle homogenate of both 6-week-old Rpt3^−/−^ and Rpt3^+/+^ mice. (F) Immunoblotting of Rpt3 protein using homogenates of tibialis anterior muscles from 2- and 6-week-old mice. (G) HE staining of tibialis anterior muscles from 2- and 6-week-old mice. Scale bar: 50 µm. (H) Changes in the components of the proteasomal complex of the tibialis anterior muscles. Rpt2 is significantly increased in Rpt3^−/−^ mice compared with its expression in Rpt3^+/+^ mice. White bars, Rpt3^+/+^; gray bars, Rpt3^−/−^. AU, arbitrary units. All quantitative data show the mean+s.e.m.; *P<0.05 (Student's *t*-test).

To investigate the effect of Rpt3 deletion on proteasomal activity in skeletal muscle tissue, a knockdown of Rpt3 by small interfering (si)RNA in C2C12 cultured myoblasts was performed. Proteasomal activity was markedly reduced in C2C12 cells after siRNA-mediated Rpt3 knockdown at 24, 48 and 72 h after transfection (Fig. 4A,B). In the immunoblotting analysis, the Rpt3 band was undetectable after siRNA treatment, indicating that the Rpt3 antibody correctly detected the Rpt3 protein (Fig. 4C).

Then, to investigate the effect of Rpt3 deletion *in vivo*, both chymotrypsin-like and trypsin-like proteasomal activities in the fast-twitch-dominant tibialis anterior muscles of 2-week-old mice were examined. The proteasomal activity in the muscle homogenate from Rpt3^−/−^ mice was significantly lower compared with that from Rpt3^+/+^ mice at 2 weeks of age (Fig. 4D). Proteasomal activity at 6 weeks of age, however, was higher in the Rpt3-deleted animals compared with Rpt3^+/+^ mice (Fig. 4E). To examine whether the detected chymotrypsin-like and trypsin-like activities were proteasome-dependent, a highly specific proteasomal inhibitor, AdaAhx_3_L_3_VS (30 µM), was added to the muscle homogenates. The proteasome-specific inhibitor significantly reduced both the chymotrypsin-like and trypsin-like proteasome activities of tibialis anterior muscles from 6-week-old Rpt3^−/−^ and Rpt3^+/+^ mice (Fig. 4E). Therefore, the increased proteasomal activity in Rpt3^−/−^ mice might not be due to the recovery or acquisition of Rpt3 because the Rpt3 protein was still markedly reduced at 6 weeks of age in Rpt3^−/−^ mice (Fig. 4F). Indeed, corresponding to the enhanced chymotrypsin-like and trypsin-like proteolytic activity, the transcription of proteasomal components in the proteolytic subunits of the 20S catalytic core, including PSMB5 with chymotrypsin-like activity and PSMB7 with trypsin-like activity, was markedly activated in the tibialis anterior muscle homogenate (supplementary material Fig. S2).

A morphological comparison of tibialis anterior muscle tissue from 2-week-old and 6-week-old Rpt3^+/+^ and Rpt3^−/−^ mice (Fig. 4G) demonstrated a clearly increased number of irregularly shaped abnormal muscle fibers in 6-week-old mice that were characterized by swelling and dislocated and enlarged nuclei. The interstitial spaces were extended, and the number of infiltrating cells was increased.

An examination of the protein components of the proteasome other than Rpt3 in tibialis anterior muscle tissue revealed increased expression of Rpt2 (also known as PSMC1) in 6-week-old Rpt3^−/−^ mice (Fig. 4H). No significant difference in the protein level of Rpt6 (also known as PSMC5), the binding partner of Rpt3, was identified in Rpt3^−/−^ mice compared with Rpt3^+/+^ mice (Fig. 4H). In addition, no significant differences in the protein levels of Rpt5, PA28α (also known as PSMC3, PSME1, respectively) or 20S proteasome proteins were noted between Rpt3^+/+^ and Rpt3^−/−^ mice (Fig. 4H).

### The transcriptional and protein levels of ubiquitin proteasomal components and the upstream pathway in Rpt3^−/−^ mice

**Fig. 5.**
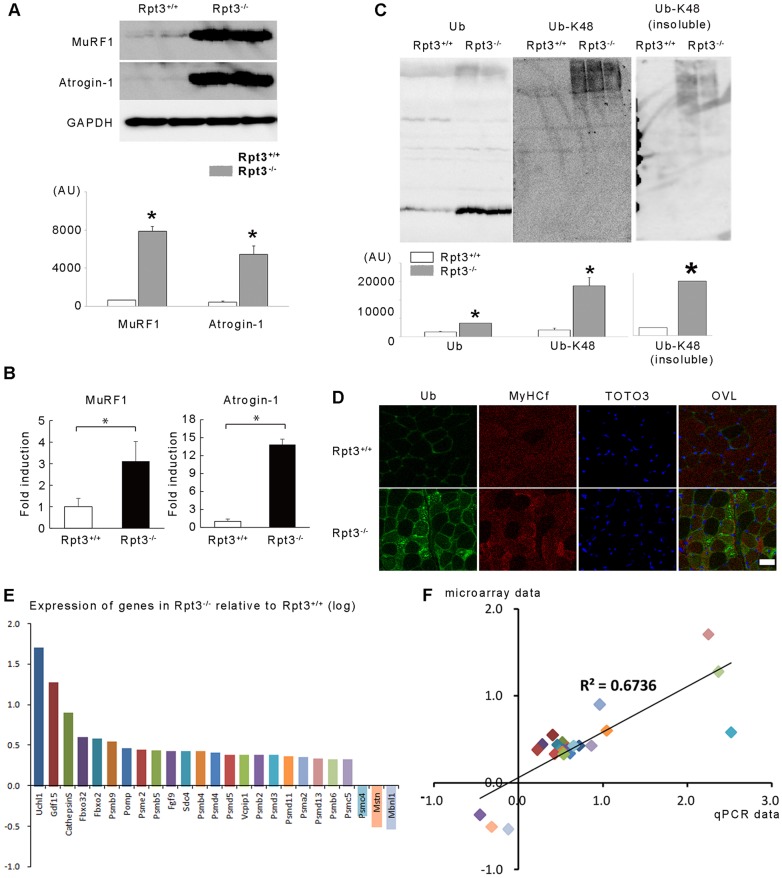
**The ubiquitin–proteasome pathway is dysregulated in the gastrocnemius muscles of Rpt3^−/−^ mice.** (A) Protein levels of the muscle-specific ubiquitin-E3-ligases MuRF1 and atrogin-1 were higher in Rpt3^−/−^ mice than in Rpt3^+/+^ mice at the age of 6 weeks. Quantitative data are also presented (*n* = 3). AU, arbitrary units. (B) Upregulation of the crucial atrophy-related and muscle-specific genes in Rpt3^−/−^ mice at the age of 6 weeks. RNA was extracted from the gastrocnemius muscles, and quantitative PCR analysis was performed in triplicate using specific primers (supplementary material Table S1). Data were normalized to the GAPDH content and expressed as the fold increase over the expression levels in Rpt3^+/+^ mice (*n* = 5). (C) The levels of ubiquitin (Ub) and high-molecular-mass ubiquitylated proteins were increased in Rpt3^−/−^ mice. Protein polyubiquitylated at the Lys48 residue (detected using an anti-Ub-K48 antibody) was also increased in Rpt3^−/−^ mice. Quantitative data are also presented (*n* = 3). Quantitative data in A–C show the mean+s.e.m.; *P<0.05 (Student's *t*-test). (D) Immunohistochemical detection of ubiquitin and MyHCf revealed the accumulation of ubiquitylated proteins, particularly in fast-twitch muscle fibers. TOTO3, nuclei; OVL, overlay. Scale bar: 50 µm. (E) Upregulation of crucial proteasome-related genes in the skeletal muscles of adult Rpt3^−/−^ mice. RNA was extracted from the gastrocnemius muscles. The *y*-axis represents the quantitative values of gene expression in Rpt3^−/−^ mice relative to Rpt3^+/+^ mice, which were transformed to log_10_ values. The value ‘0’ indicates equal gene expression between Rpt3^−/−^ and Rpt3^+/+^ mice. Of the several proteasome-related genes that were measured, the expression of PSMC4 (Rpt3) was inhibited in Rpt3^−/−^ mice compared with that of Rpt3^+/+^ mice. Quantitative PCR analysis was performed in triplicate using specific primers (supplementary material Table S1). (F) Relative expression levels, normalized to β-actin, were well correlated between the microarray data and the quantitative PCR analysis (*R*^2^ = 0.6736).

Because the activity of the ubiquitin proteasomal pathway depends upon an upstream transcriptional program that requires the activation of a subset of atrophy-related genes, or atrogenes (Lecker et al., 2004), the expression of atrogenes involved in the ubiquitin–proteasomal pathway in the fast-twitch-dominant gastrocnemius muscle of Rpt3^−/−^ mice was examined. Indeed, the protein and transcriptional levels of two atrophy-related ubiquitin E3 ligases, MuRF1 and atrogin-1, were markedly increased in Rpt3^−/−^ mouse gastrocnemius muscle compared with that of Rpt3^+/+^ mice (Fig. 5A,B). The levels of ubiquitin and polyubiquitylated protein at the Lys48 residue were also markedly increased (Fig. 5C). An immunohistochemical examination also demonstrated a marked increase or accumulation of polyubiquitylated proteins in the fast-twitch muscle fibers of the gastrocnemius muscle from Rpt3^−/−^ mice (Fig. 5D).

To obtain an overall view of the gene expression profiles in Rpt3^−/−^ skeletal muscle, microarray and real-time PCR analyses of gastrocnemius muscle tissue were performed (Fig. 5E,F; primers are listed in supplementary material Table S1). Proteasome-related genes were upregulated in Rpt3^−/−^ mice; a summary of the top ten networks identified by Ingenuity pathway analysis of the microarray data revealed that ‘connective tissue proliferation’, ‘cellular development’ and ‘protein synthesis’ were all affected in Rpt3^−/−^ mice (supplementary material Table S2). The microarray data are available online (GEO; http://www.ncbi.nlm.nih.gov/geo/) under the accession number GSE34896. A pathway analysis using DAVID online software provided by the National Institute of Allergy and Infectious Diseases (NIAID), NIH (http://david.abcc.ncifcrf.gov/home.jsp) was performed. A KEGG pathway analysis using the list of genes that were differentially expressed over 1.5-fold compared with their expression in Rpt3^+/+^ mice is shown in supplementary material Table S3. Proteasome- and lysosome-related genes were significantly upregulated, as was the MAPK pathway in Rpt3^−/−^ mice (supplementary material Table S3). The protein level of phosphorylated p38 was increased in the gastrocnemius muscle of the Rpt3^−/−^ mice (Fig. 7A).

**Fig. 6.**
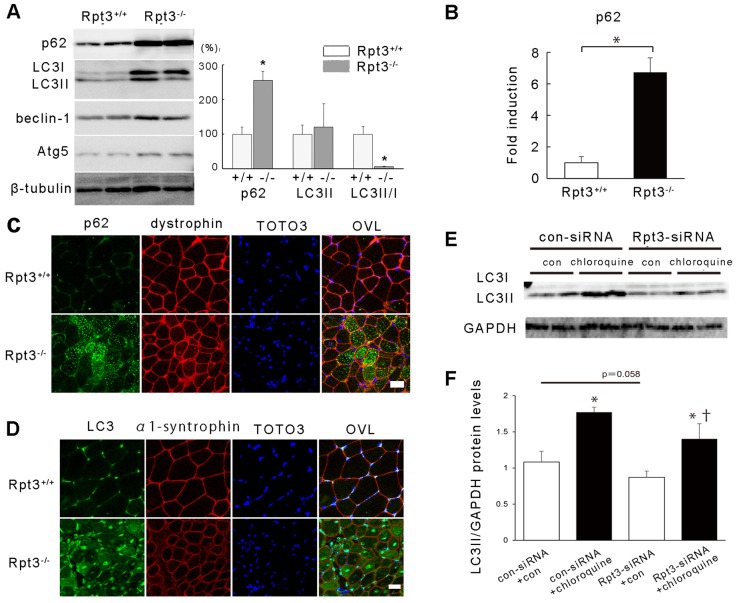
**Autophagy is reduced in the gastrocnemius muscles of Rpt3^−/−^ mice.** (A) Immunoblotting revealed increased levels of p62, LC3, beclin-1 and Atg5 proteins in Rpt3^−/−^ mice at the age of 6 weeks. Quantitative data are also presented (*n* = 3). Note that p62 was increased and the LC3II∶LC3I ratio was decreased in Rpt3^−/−^ mice. (B) Upregulation of p62 mRNA in Rpt3^−/−^ mice at the age of 6 weeks. RNA was extracted from the gastrocnemius muscles, and quantitative PCR analysis was performed in triplicate using specific primers (supplementary material Table S1). Data were normalized to the GAPDH content and expressed as the fold increase over the level of expression in Rpt3^+/+^ mice (*n* = 5). (C) Immunohistochemical examination revealed p62-positive myofibers in Rpt3^−/−^ mice at the age of 6 weeks. Scale bar: 50 µm. (D) Immunohistochemical examination revealed LC3-positive myofibers in Rpt3^−/−^ mice at the age of 6 weeks. TOTO3, nuclei; OVL, overlay. Scale bar: 50 µm. (E) Autophagy flux was investigated using C2C12 cells. Representative immunoblotting showing autophagy flux assay with reduced LC3II levels following Rpt3 knockdown under chloroquine inhibition (*n* = 4/treatment). con, control. (F) The protein level of LC3II was significantly different between vehicle- and chloroquine-treated samples (*P<0.05). The protein level of LC3II was significantly different between samples treated with both Rpt3 siRNA and chloroquine and those treated with control siRNA and chloroquine (^†^*P*<0.05). The protein level of LC3II was not significantly different between samples treated with control siRNA without chloroquine those treated with Rpt3 siRNA without chloroquine (*P* = 0.058). Quantitative data in A,B,F show the mean+s.e.m.; *P<0.05 (Student's *t*-test).

The autophagy pathway is another important mechanism that is responsible for protein degradation and processing within cells. The protein p62 (also known as SQSTM1) has a role in the aggregation of intracellular ubiquitin-related protein (Komatsu et al., 2007). LC3 (also known as MAP1LC3A and MAP1LC3B) is a post-translational modifier that is essential for autophagosome formation. The protein and transcriptional levels of p62 were markedly increased in Rpt3^−/−^ mouse gastrocnemius muscle compared with the same muscle from Rpt3^+/+^ mice (Fig. 6A,B). There was also an increase in LC3II and a marked decrease in the LC3II∶LC3I ratio in Rpt3^−/−^ mice (Fig. 6A). Immunohistochemical analyses detected a marked increase in the amount of p62 and LC3 in the myofibers of Rpt3^−/−^ mutant animals (Fig. 6C,D).

The influence of Rpt3 deficiency on autophagy flux was examined in cultured C2C12 cells. The administration of the lysosome inhibitor chloroquine to C2C12 cells with or without Rpt3 siRNA resulted in an increase in LC3II protein (Fig. 6E,F), but the increase was attenuated in the Rpt3-knockdown C2C12 cells.

**Fig. 7.**
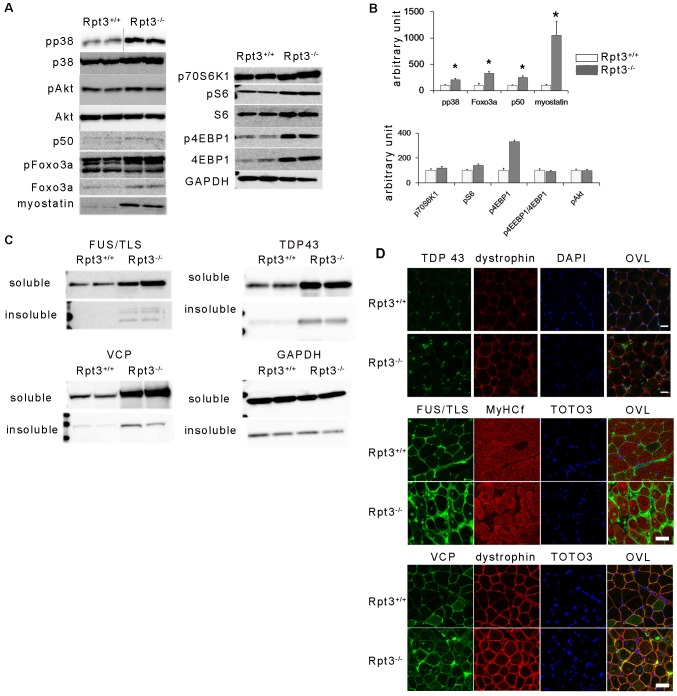
**Catabolic pathways in the gastrocnemius muscles of Rpt3^−/−^ mice.** (A) Immunoblotting analysis for the detection of muscle atrophy-related signaling pathway components. (B) Quantification of the data from A. The amount of phosphorylated p38 (pp38) was also increased in Rpt3^−/−^ mice. No apparent differences were observed in the levels of Akt and phosphorylated (p)Akt. Foxo3a, p50, and myostatin were increased. Phosphorylated (p)Foxo3a, p70S6K1, phosphorylated (p)S6, S6, phosphorylated (p)4EBP1, 4EBP1 and GAPDH were also present. The ratios of phosphorylated S6∶S6 and phosphorylated 4EBP1∶4EBP1 were not altered, whereas the total amount of phosphorylated 4EBP1 was increased. Data show the mean+s.e.m.; *P<0.05 (Student's *t*-test). (C) Proteins related to RNA metabolism accumulated in myofibers. Expression of the FUS/TLS, VCP and TDP-43 proteins was increased in Rpt3^−/−^ mice, especially in the insoluble fractions. (D) Immunohistochemistry of TDP-43, FUS/TLS and VCP revealed that the amount of these markers localizing within myonuclei in Rpt3^−/−^ mice was increased. TOTO3, nuclei; OVL, overlay. Scale bars: 20 µm (upper panel), 50 µm (middle and lower panels).

The levels of phosphorylated p38, phosphorylated Foxo3a, Foxo3a, p50 (also known as NFKB1) and myostatin were increased in the gastrocnemius muscle of Rpt3^−/−^ mice (Fig. 7A,B). Although the protein level of Foxo3a was significantly higher in Rpt3^−/−^ mice, no difference in the ratio of Foxo3a to its phosphorylated form was detected between Rpt3^−/−^ and Rpt3^+/+^ mice. The protein levels of p70S6K1 and the ratios of phosphorylated S6 (also known as RPS6), 4EBP1 and Akt were similar in gastrocnemius muscles from both Rpt3^+/+^ and Rpt3^−/−^ mice (Fig. 7A,B). Additionally, the total amount of 4EBP1 and phosphorylated 4EBP1 was increased. Therefore, the downregulation of protein synthesis did not likely contribute to muscle atrophy or the degeneration of Rpt3^−/−^ muscle in this study.

Several diseases are characterized by abnormal protein aggregates in myofibers. The expression of TDP-43 and fused in sarcoma/translocated in liposarcoma (FUS/TLS) has been observed in muscle biopsy samples from patients with sporadic inclusion body myositis (sIBM) (Weihl and Pestronk, 2010; Yamashita et al., 2013). The amounts of these proteins differed between Rpt3^+/+^ and Rpt3^−/−^ mice, particularly in the insoluble fraction, when evaluated by immunoblotting (Fig. 7C). Immunohistochemical examination revealed enhanced staining of TDP-43, FUS/TLS and VCP in the myonuclei of gastrocnemius muscle from Rpt3^−/−^ mice (Fig. 7D).

## DISCUSSION

In this study, we report that the fast-twitch muscle-specific deletion of a crucial proteasomal gene, *Rpt3*, resulted in profound muscle growth defects and a decrease in force production in mice with the accumulation of abnormal proteins. To the best of our knowledge, our study is the first to examine the role of the proteasomal system in mammalian skeletal muscle using a loss-of-function strategy. Contrary to our hypothesis and previous studies using proteasome inhibitors on dystrophic mice or myostatin-defective mice, in which muscle hypertrophy was demonstrated (McPherron et al., 1999), the specific deletion of the proteasome component Rpt3 led to a significant loss of muscle mass with premature death and significantly reduced physical activity.

To generate a skeletal-muscle-specific Rpt3 conditional knockout, we used Cre under the control of the Mlc1f promoter, with which we could delete Rpt3 only in fast-twitch muscle fibers. We first used α1 skeletal muscle actin promoter (ACTA1)-Cre transgenic mice so that Cre would be expressed without fiber-type specificity; however, this deletion resulted in embryonic lethality. The skeletal muscle creatine kinase (MCK)-Cre transgenic, which is often used to establish muscle-specific loss of function, was also a candidate, but we did not use this line because the MCK-Cre transgenic is known also to induce Cre in cardiac muscle tissue. Therefore, although we failed to demonstrate the histological distribution of Rpt3 in muscle tissue, most likely owing to the non-specific binding of the Rpt3 polyclonal antibody, the expression of Rpt3 in slow-twitch fibers might not have been affected.

In this study, because there was no difference in the size of slow-twitch fibers between Rpt3^+/+^ and Rpt3^−/−^ mice and because the proportion of slow-twitch muscle was increased in the gastrocnemius muscle of Rpt3^−/−^ mice at older age, we assume that there was a selective reduction of fast-twitch fibers in the gastrocnemius muscle. The premature death observed in Rpt3^−/−^ mice might be a consequence of impaired fast-twitch fibers. The movement of Rpt3^−/−^ mice was slow with a significantly reduced step pace. The impaired movement might have limited their access to food, but the precise cause of premature death remains unknown. However, not all fast-twitch fibers were lost because the tibialis anterior muscle, of which >99% is fast-twitch fibers (Augusto et al., 2004), maintained fast-twitch fibers even at 6 weeks of age.

Proteasomal activity was significantly reduced in the whole-muscle preparation at 2 weeks of age as measured both by chymotrypsin-like and trypsin-like activity in the Rpt3^−/−^ mice. The fact that Rpt3 knockdown in C2C12 myoblasts led to a >80% reduction in proteasomal activity suggests that at the single fast-twitch fiber level, proteasomal activity was largely abrogated in Rpt3^−/−^ mice. The reason that the reduction in proteasomal activity in tibialis anterior muscle remained at only ∼20% *in vivo* at the age of 2 weeks might be due to the excision efficiency of the Mlc1f-promoter-driven Cre, which is ∼40–50% (Bothe et al., 2000). Therefore, some myonuclei that were positive for *Rpt3* might have remained. Additionally, non-muscle cells, such as inflammatory cells, might have contributed to the proteasomal activity.

The results obtained in this study markedly contrasted with the results obtained in previous studies using the proteasome inhibitor MG-132, in which the administration of MG-132 significantly improved the dystrophic phenotype (Carmignac et al., 2011). MG-132 also improved the dystrophic phenotype in a model of dystrophin deficiency (Bonuccelli et al., 2003; Winder et al., 2011). The administration of MG-132 might have effects on all tissues and organs. Specifically, MG-132 is known to protect IκB, which inhibits NFκB, from proteasomal degradation in inflammatory cells. Thus, an attenuated inflammatory process might have favored mice with a dystrophin deficiency (Lee and Goldberg, 1998). This was one of our strong motivations to establish a skeletal muscle-specific deletion of the Rpt3 component of the proteasome complex without affecting non-muscle cell types, such as inflammatory cells, in order to achieve a better understand the role of the ubiquitin–proteasome pathway in skeletal muscle cells.

Interestingly, although the proteasomal activity was significantly suppressed in Rpt3^−/−^ fast-twitch dominant skeletal muscles at 2 weeks of age, a marked enhancement, rather than a mere recovery, in trypsin-like and chymotrypsin-like proteasomal activity was observed in the skeletal muscle at 6 weeks of age. Indeed, the transcription of the proteasomal components PSMB5 with chymotrypsin-like activity and PSMB7 with trypsin-like activity, which are proteolytic subunits of the 20S catalytic core, was markedly increased in Rpt3^−/−^ mice at 6 weeks of age. Some possibilities might explain the apparent enhancement in proteolytic activity, and these issues need to be further addressed. First, the involvement of proteolytic activity from a non-proteasomal source, such as lysosomes, is not likely because the proteolytic activity in the muscle homogenate of the tibialis anterior muscle was sensitive to a highly specific proteasome inhibitor. Second, because the tibialis anterior muscle is composed mostly of fast-twitch fibers and there was no fiber-type switching in Rpt3^−/−^ mice, the possibility of the contribution of intact proteasomes from slow-twitch fibers should be almost negligible (supplementary material Fig. S1). One of the more plausible explanations for the increase in proteasomal proteolytic activity at an older age is the upregulation of the proteolysis pathways that counteract the reduced degradation of ubiquitylated proteins. An increased amount of the ubiquitin E3 ligases MuRF1 and atrogin-1 in Rpt3^−/−^ gastrocnemius muscle might also have contributed to the accumulation of ubiquitylated proteins, but this result could also be a part of a counteraction against impaired protein degradation through proteasomes. Although no change was found in the ratio between Foxo3a and its phosphorylated form, the amount of Foxo3, which transcriptionally activates MuRF1 and atrogin-1 expression, was higher in Rpt3^−/−^ compared with Rpt3^+/+^ mice. The apparent myocellular effort of counter-regulation against impaired protein degradation, however, did not seem to be effective in the restoration of effective protein degradation because there was a remarkable accumulation of ubiquitylated proteins in the muscle tissue of Rpt3^−/−^ mice at 6 weeks of age.

One of the aims of this study was to investigate the influence of autophagy activity when proteasomal function was impaired. In Rpt3^−/−^ muscle tissue, the initial steps to transfer ubiquitylated proteins to the autophagy pathway seem to be enhanced. Ubiquitin-binding p62 was markedly increased by more than twofold in Rpt3^−/−^ gastrocnemius muscle, and immunostaining of p62 was detected within Rpt3^−/−^ muscle fibers. Beclin-1 and Atg-5 that are involved in isolation membrane formation were also increased in Rpt3^−/−^ tissue. LC3I, which is involved in the initiation of autophagosome formation, was markedly increased in Rpt3^−/−^ gastrocnemius muscle. To this extent, the autophagy pathway seems to be enhanced. Although the LC3I protein level was markedly increased in Rpt3^−/−^ gastrocnemius muscle homogenate at 6 weeks of age, the conversion to LC3II (LC3II∶LC3I) was significantly reduced. Therefore, the progression of autophagolysosomal formation seems to be impaired. An independent cellular study using cultured C2C12 myotubes revealed that Rpt3 knockdown resulted in the suppression of LC3 turnover, suggesting a contribution of the proteasomal proteolytic process in autophagy progression. Taken together, a cellular effort appears to compensate for impaired proteasomal proteolysis caused by the absence of Rpt3 in the proteasomal complex by delivering ubiquitylated proteins to the autophagy pathway, but autophagy seems to be impaired at autophagosome formation by an unknown but proteasomal-activity-dependent mechanism. The lack of increase in the number of autophagosomes as revealed by electron microscopy might correspond to this observation. Increased myostatin expression might also have contributed to the enhanced expression of autophagy proteins (Lee et al., 2011).

The increase in p62 and the formation of inclusion bodies observed in Rpt3^−/−^ mice was previously reported as a pathophysiological condition induced by a deficiency in autophagy (Komatsu et al., 2007). In addition to muscle atrophy, we also observed a marked increase in proteins associated with myopathies and neurodegenerative diseases, such as VCP, TDP-43 and FUS/TLS, within the muscle fibers of Rpt3^−/−^ mice. Indeed, VCP is a molecular adapter that binds to the ubiquitin of a ubiquitylated protein and autophagosome-specific proteins (Tresse et al., 2010). The concomitant increase in VCP and p62 seems to be reasonable. These proteins might be cooperating in transferring accumulated ubiquitylated proteins to the selective autophagy pathway. The accumulation of TDP-43 within the nuclei of Rpt3^−/−^ myofibers is another interesting finding. TDP-43 is an RNA-binding protein suggested to be required for the maintenance of the autophagy pathway by stabilizing *Atg7* mRNA (Bose et al., 2011). A marked increase in TDP-43 might further support the compensatory activation of the selective autophagy pathway due to impaired proteasomal activity. The FUS/TLS protein also has RNA binding capability, has a transcriptional activation property (Fiesel and Kahle, 2011) and is enhanced in Rpt3^−/−^ muscle myonuclei; however, the distinct role of this protein in the Rpt3^−/−^ pathology is unknown. TDP-43, FUS/TLS and p62 are integral components of sIBM inclusions, with p62 immunoreactivity being particularly specific and strong, and these proteins can be used as disease markers for sIBM (Nogalska et al., 2009; Salajegheh et al., 2009). Our finding in this study suggests that the pathological mechanism might be similar to that of sIBM, although electron microscopic analysis of Rpt3^−/−^ mice did not reveal myelin-like structures similar to those observed in patients with sIBM. Proteasomal activity in the muscles of patients with sIBM is reported as either activated or suppressed (Ferrer et al., 2005; Fratta et al., 2005); thus, multiple pathological mechanisms might be involved. Our findings might partly explain the pathology of sIBM with suppressed proteasomal activity.

Impaired autophagy is known to induce skeletal myofiber degeneration and muscle weakness (Masiero et al., 2009; Raben et al., 2008). Using the same Mlcf-Cre promoter, Masiero et al. generated fast-twitch muscle-specific *Atg7*-knockout mice to disrupt the autophagy pathway in skeletal muscle (Masiero et al., 2009). We found similar myopathic changes in the Rtp3^−/−^ mice as in the Atg7^−/−^ mice, but the extent of muscle wasting or failure in muscle development was apparently more severe in the Rpt3^−/−^ mice. The body weight of Atg7^−/−^ mice was reported to be slightly lower than that of the control mice, whereas Rpt3^−/−^ mice exhibited a marked impairment in body weight growth mainly due to impaired muscle growth. We found not only weakness in muscle strength but also a serious gait disturbance due to muscle weakness. Premature death was observed in Rpt3^−/−^ mice but not in Atg7^−/−^ mice. Considering that the same fast-twitch muscle cells were affected in these conditional knockout mice, we conclude that the proteasomal pathway has a greater impact on maintaining the homeostasis of skeletal muscle tissue.

Referring to the fundamental role of autophagy, the autophagy process is promoted when cells are cultured under starved conditions without amino acids in the culture medium (Kuma and Mizushima, 2010). Our study suggests that both the ubiquitin proteasome and autophagy systems are required to maintain myocellular homeostasis and integrity. The protein and organelle degradation by both autophagy and ubiquitin proteasome systems might provide resources, such as oligopeptides and amino acids, for maintaining cellular integrity in skeletal muscle tissue (Bonaldo and Sandri, 2013). Therefore, the Rpt3 deficiency might have resulted in a far more serious condition that deprives the cells of two major paths of pooling resources for cellular maintenance. We hypothesize that the skeletal muscle Rpt3 deficiency might have led to blocking the cellular ‘recycling system’ that is essential to the maintenance of skeletal muscle fibers, and this question needs to be further examined. In *Drosophila*, the progressive accumulation of protein aggregates is a characteristic of aging in skeletal muscle (Demontis and Perrimon, 2010). Using the conditional expression of a mutant proteasome β subunit in *Drosophila*, Haas et al. reported that the ubiquitin proteasome system is required for the acute maintenance of muscle and neuromuscular junction architecture (Haas et al., 2007). Taken together, these results suggest that basal, appropriately balanced proteasomal activity has a beneficial role in the control of muscle mass during muscle growth.

## MATERIALS AND METHODS

### Generation of muscle-specific Rpt3-knockout mice

Mice bearing a floxed Rpt3 allele (Tashiro et al., 2012) (Rpt3 f/f) were crossed with transgenic mice expressing Cre under the control of either a myosin light chain 1 fast promoter (MLC1f-Cre) (Bothe et al., 2000) or an α1 skeletal muscle actin promoter (ACTA1-Cre) (Miniou et al., 1999). Genomic DNA isolated from mice bearing the Cre allele or Rpt3-lox was subjected to standard PCR analysis. The animals were provided access to food and drinking water *ad libitum* and were euthanized by cervical dislocation under anesthesia. The tibialis anterior and gastrocnemius muscles were subsequently excised for analysis. All of the experimental protocols and procedures were approved by the Animal Committee of the Tohoku University School of Medicine Ethics Committee (animal 2011-234).

### Mouse tissue preparation

Body and wet muscle weights were determined. Tibialis anterior and gastrocnemius muscles were collected individually using standard dissection methods and cleared of excess fat, connective tissue and tendons, and subjected to further preparation and analyses. The origins of muscle samples either from tibialis anterior or gastrocnemius muscle is described in each figure legend. Some portions of the muscles were frozen in isopentane cooled with liquid nitrogen for histological and immunohistochemical analysis, and the other muscle portions were frozen directly in liquid nitrogen and stored at −80°C for RNA isolation or protein extraction.

### Antibodies

Antibodies against the following proteins were obtained from Cell Signaling Technology: Akt, phosphorylated Akt (Ser473), phosphorylated p38, p38, ubiquitin, protein polyubiquitylated at the Lys48 residue (Ub-K48), Atg5, beclin-1, Hsp90, p70S6K1, phosphorylated S6 (Ser240/244), S6, phosphorylated 4EBP1 and 4EBP1. Antibodies against Rpt2, Rpt3, Rpt5, PA28α and 20S were purchased from Enzo. MuRF1 and atrogin-1 antibodies were purchased from ECM Biosciences. Calsequestrin antibodies were purchased from Abcam. Antibodies against fast, slow, developmental and neonatal myosin heavy chain and antibodies against dystrobrevin, dystrophin, α-dystroglycan, α-sarcoglycan and caveolin-3 were obtained from Novocastra. Serca and laminin antibodies were obtained from Sigma. We also used antibodies against GAPDH (Santa Cruz Biotechnology), LC3 (Novus Biological and nanoTools Antikörpertechnik), p62 (PROGEN Biotechnik), TDP-43 (Proteintech), FHL1 (Abcam), VCP (BD Biosciences), FUS/TLS (Bethyl Laboratories), α1-syntrophin (Thermo Fisher Scientific) and neuronal nitric oxide synthase (Invitrogen).

### Proteasome activity

Proteasome activity was assessed using Proteasome-Glo^TM^ Assay kit (Promega) following the manufacturer's instruction. The trypsin-like and chymotrypsin-like activity assays were conducted using skeletal muscle homogenates in a total volume of 100 µl in opaque 96-well plates. For the assays, 120 µg of protein was added to assay buffer containing 20 mM Tris-HCl (pH 7.2), 0.1 mM EDTA, 5 mM ATP, 1 mM β-mercaptoethanol, 20% glycerol and 0.04% Nonidet P40. The individual proteasome reagents were added separately, and 30 min later, the luminescence was recorded as relative light units on a Varioskan luminometer (Thermo Scientific). Each sample was measured in triplicate.

In addition, to evaluate proteasome activity more precisely, dual measurements with or without the addition of 30 µM of the irreversible and highly specific proteasomal inhibitor adamantine-acetyl-(6-aminohexanoyl)_3_-(leucinyl)_3_-vinyl-(methyl)-sulfone (AdaAhx_3_L_3_VS, Calbiochem, catalog number 114802) were carried out (Kessler et al., 2001).

### Creatine kinase measurement

Blood (200 µl) was collected in Eppendorf tubes using cardiac puncture under deep anesthesia, and it was allowed to clot at room temperature prior to centrifugation and serum collection. Creatine kinase was measured using a standard spectrophotometric method according to the manufacturer's instructions. The data are expressed as units per liter.

### Functional tests

Forearm grip strength was assessed in 8-week-old mice using a grip strength meter (GPM-100, MelQuest) according to the manufacturer's instructions. An investigator blinded to the treatment conditions recorded three successful forelimb strength measurements (*n* = 5) in the morning. The average grip strength measurement obtained each day was used for subsequent analysis. Motor endurance was measured using a round cage (RS-204-5, Kori-Seiki). The number of rotations per day was recorded, and the average number of rotations was calculated on three consecutive days (*n* = 5).

### Step pace analysis

After conditioning runs, the plantar surface of the hind paws was impregnated with black printing paint and each mouse walked with office copier paper on the base. Four prints of each foot were recorded on the length of the paper used. The distance of the print length was measured as step pace (mm). The walking track test was performed at 6 weeks of age.

### Microarray analysis and real-time PCR

Total RNA was isolated using an RNeasy kit (Qiagen). The RNA was subjected to microarray analysis using a Codelink mouse whole-genome bioarray according to the manufacturer's instructions. We performed KEGG pathway analysis using the list of genes that were differentially expressed over 1.5 times from Rpt3^+/+^ mice (supplementary material Table S3).

For real-time PCR, first-strand cDNA synthesis was performed using oligo-dT primers. The expression levels of selected genes were analyzed using the Bio-Rad CFX96 system according to the manufacturer's instructions and quantitative PCR analysis was performed in triplicate using specific primers (supplementary material Table S1).

### Immunohistochemistry

Cryosections of muscle tissue (10-µm thickness) were cut from the middle portion of the muscle belly to obtain the largest myofiber diameter, placed on poly-L-lysine-coated slides, air-dried, post-fixed in acetone at −20°C and pre-incubated in phosphate-buffered saline (PBS) containing 5% goat serum and 1% bovine serum albumin (BSA) for 30 min at room temperature. The primary antibodies were applied overnight at 4°C. Following incubation with the appropriate secondary antibodies, the mounted sections were observed using an Olympus confocal microscope.

### Electron microscopy

The skeletal muscles were fixed using 4% paraformaldehyde and 2% glutaraldehyde for conventional electron microscopy. The samples were post-fixed with 1% OsO_4_, embedded in Epon epoxy resin and sectioned.

### Immunoblotting analysis

The total skeletal muscle protein was extracted from the tibialis anterior muscles and gastrocnemius muscles for immunoblotting analysis. We described in the figure legends which muscles we used. We used the Bradford method to determine the total protein concentration. The protein fractions were then extracted with a reducing sample buffer containing 2.3% SDS, 70 mM Tris-HCl, 5% β-mercaptoethanol, and Complete Protease Inhibitor Cocktail (Roche). Proteins (30 µg per lane) were separated on a 10–20% gradient SDS-polyacrylamide gel and subsequently transferred to a polyvinylidene difluoride membrane (Millipore) at 240 mA for 1 h. The membrane was then incubated with primary antibody. Specific signals were detected using the enhanced chemiluminescence method (GE Healthcare), as described previously (Suzuki et al., 2007). Densitometry was performed using ImageJ software (National Institutes of Health). For the fractionation of soluble and insoluble proteins, a 6 M urea solution was used.

### C2C12 experiments

Mouse C2C12 myoblasts were cultured under standard conditions (37°C under a humidified atmosphere containing 5% CO_2_) in high-glucose DMEM supplemented with 10% fetal bovine serum and 100 µg/ml penicillin-streptomycin solution. The cells were transfected with 30 nM siRNA using Lipofectamine RNAiMAX (Invitrogen). To examine the proteasomal activity, C2C12 cells were harvested in the aforementioned assay buffer using a cell scraper at 24, 48 and 72 h after the transfection. After centrifugation (13,000 ***g*** for 15 min at 4°C), 100 µg of the cleared protein sample was incubated in the presence of 40 µM of fluorogenic substrate in reaction buffer. Autophagy flux was evaluated using the administration of 50 µM chloroquine (Sigma-Aldrich) as described previously (Mizushima et al., 2010).

### Statistical analysis

Significant differences were determined using either Student's unpaired *t*-test or the Mann–Whitney *U*-test. All data are expressed as the mean±s.e.m. Statistical significance was defined as *P*<0.05.

### Supplemental data

The microarray data have been deposited into the Gene Expression Omnibus (GEO; http://www.ncbi.nlm.nih.gov/geo/) under the accession number GSE34896. KEGG pathway analysis was performed using the list of genes differentially expressed over 1.5 times from Rpt3^+/+^ mice (supplementary material Table S3).

## Supplementary Material

Supplementary Material

## References

[b1] Augusto V. Padovani C. R. Campos G. E. R. (2004). Skeletal muscle fiber types in C57BL6J mice. Braz. J. Morphol. Sci. 21, 89–94.

[b2] Bedford L. Hay D. Devoy A. Paine S. Powe D. G. Seth R. Gray T. Topham I. Fone K. Rezvani N. (2008). Depletion of 26S proteasomes in mouse brain neurons causes neurodegeneration and Lewy-like inclusions resembling human pale bodies. J. Neurosci. 28, 8189–8198 10.1523/JNEUROSCI.2218-08.2008 18701681 PMC6670564

[b3] Bonaldo P. Sandri M. (2013). Cellular and molecular mechanisms of muscle atrophy. Dis. Model. Mech. 6, 25–39 10.1242/dmm.010389 23268536 PMC3529336

[b4] Bonuccelli G. Sotgia F. Schubert W. Park D. S. Frank P. G. Woodman S. E. Insabato L. Cammer M. Minetti C. Lisanti M. P. (2003). Proteasome inhibitor (MG-132) treatment of mdx mice rescues the expression and membrane localization of dystrophin and dystrophin-associated proteins. Am. J. Pathol. 163, 1663–1675 10.1016/S0002-9440(10)63523-7 14507673 PMC1868305

[b5] Bose J. K. Huang C. C. Shen C. K. (2011). Regulation of autophagy by neuropathological protein TDP-43. J. Biol. Chem. 286, 44441–44448 10.1074/jbc.M111.237115 22052911 PMC3247982

[b6] Bothe G. W. Haspel J. A. Smith C. L. Wiener H. H. Burden S. J. (2000). Selective expression of Cre recombinase in skeletal muscle fibers. Genesis 26, 165–166 10.1002/(SICI)1526-968X(200002)26:2<165::AID-GENE22>3.0.CO;2-F 10686620

[b7] Braun T. Gautel M. (2011). Transcriptional mechanisms regulating skeletal muscle differentiation, growth and homeostasis. Nat. Rev. Mol. Cell Biol. 12, 349–361 10.1038/nrm3118 21602905

[b8] Cai D. Frantz J. D. Tawa N. E. Jr Melendez P. A. Oh B. C. Lidov H. G. Hasselgren P. O. Frontera W. R. Lee J. Glass D. J. (2004). IKKbeta/NF-kappaB activation causes severe muscle wasting in mice. Cell 119, 285–298 10.1016/j.cell.2004.09.027 15479644

[b9] Carmignac V. Quéré R. Durbeej M. (2011). Proteasome inhibition improves the muscle of laminin α2 chain-deficient mice. Hum. Mol. Genet. 20, 541–552 10.1093/hmg/ddq499 21084425

[b10] Demontis F. Perrimon N. (2010). FOXO/4E-BP signaling in Drosophila muscles regulates organism-wide proteostasis during aging. Cell 143, 813–825 10.1016/j.cell.2010.10.007 21111239 PMC3066043

[b11] Ding W. X. Ni H. M. Gao W. Yoshimori T. Stolz D. B. Ron D. Yin X. M. (2007). Linking of autophagy to ubiquitin-proteasome system is important for the regulation of endoplasmic reticulum stress and cell viability. Am. J. Pathol. 171, 513–524 10.2353/ajpath.2007.070188 17620365 PMC1934546

[b12] Ferrer I. Carmona M. Blanco R. Moreno D. Torrejón-Escribano B. Olivé M. (2005). Involvement of clusterin and the aggresome in abnormal protein deposits in myofibrillar myopathies and inclusion body myositis. Brain Pathol. 15, 101–108 10.1111/j.1750-3639.2005.tb00504.x 15912881 PMC8095801

[b13] Fiesel F. C. Kahle P. J. (2011). TDP-43 and FUS/TLS: cellular functions and implications for neurodegeneration. FEBS J. 278, 3550–3568 10.1111/j.1742-4658.2011.08258.x 21777389

[b14] Fratta P. Engel W. K. McFerrin J. Davies K. J. Lin S. W. Askanas V. (2005). Proteasome inhibition and aggresome formation in sporadic inclusion-body myositis and in amyloid-beta precursor protein-overexpressing cultured human muscle fibers. Am. J. Pathol. 167, 517–526 10.1016/S0002-9440(10)62994-X 16049336 PMC1603556

[b15] Glass D. J. (2010). Signaling pathways perturbing muscle mass. Curr. Opin. Clin. Nutr. Metab. Care 13, 225–229 10.1097/MCO.0b013e32833862df 20397318

[b16] Grumati P. Coletto L. Sabatelli P. Cescon M. Angelin A. Bertaggia E. Blaauw B. Urciuolo A. Tiepolo T. Merlini L. (2010). Autophagy is defective in collagen VI muscular dystrophies, and its reactivation rescues myofiber degeneration. Nat. Med. 16, 1313–1320 10.1038/nm.2247 21037586

[b17] Haas K. F. Woodruff E. 3rd Broadie K. (2007). Proteasome function is required to maintain muscle cellular architecture. Biol. Cell 99, 615–626 10.1042/BC20070019 17523916 PMC2712885

[b18] Jagoe R. T. Goldberg A. L. (2001). What do we really know about the ubiquitin-proteasome pathway in muscle atrophy? Curr. Opin. Clin. Nutr. Metab. Care 4, 183–190 10.1097/00075197-200105000-00003 11517350

[b19] Kaneko T. Hamazaki J. Iemura S. Sasaki K. Furuyama K. Natsume T. Tanaka K. Murata S. (2009). Assembly pathway of the Mammalian proteasome base subcomplex is mediated by multiple specific chaperones. Cell 137, 914–925 10.1016/j.cell.2009.05.008 19490896

[b20] Kessler B. M. Tortorella D. Altun M. Kisselev A. F. Fiebiger E. Hekking B. G. Ploegh H. L. Overkleeft H. S. (2001). Extended peptide-based inhibitors efficiently target the proteasome and reveal overlapping specificities of the catalytic beta-subunits. Chem. Biol. 8, 913–929 10.1016/S1074-5521(01)00069-2 11564559

[b21] Komatsu M. Waguri S. Koike M. Sou Y. S. Ueno T. Hara T. Mizushima N. Iwata J. Ezaki J. Murata S. (2007). Homeostatic levels of p62 control cytoplasmic inclusion body formation in autophagy-deficient mice. Cell 131, 1149–1163 10.1016/j.cell.2007.10.035 18083104

[b22] Kuma A. Mizushima N. (2010). Physiological role of autophagy as an intracellular recycling system: with an emphasis on nutrient metabolism. Semin. Cell Dev. Biol. 21, 683–690 10.1016/j.semcdb.2010.03.002 20223289

[b23] Lecker S. H. Jagoe R. T. Gilbert A. Gomes M. Baracos V. Bailey J. Price S. R. Mitch W. E. Goldberg A. L. (2004). Multiple types of skeletal muscle atrophy involve a common program of changes in gene expression. FASEB J. 18, 39–51 10.1096/fj.03-0610com 14718385

[b24] Lee D. H. Goldberg A. L. (1998). Proteasome inhibitors: valuable new tools for cell biologists. Trends Cell Biol. 8, 397–403 10.1016/S0962-8924(98)01346-4 9789328

[b25] Lee J. Y. Hopkinson N. S. Kemp P. R. (2011). Myostatin induces autophagy in skeletal muscle in vitro. Biochem. Biophys. Res. Commun. 415, 632–636 10.1016/j.bbrc.2011.10.124 22079631

[b26] Li H. Chen Q. Liu F. Zhang X. Li W. Liu S. Zhao Y. Gong Y. Yan C. (2013). Unfolded protein response and activated degradative pathways regulation in GNE myopathy. PLoS ONE 8, e58116 10.1371/journal.pone.0058116 23472144 PMC3589370

[b27] Lyons G. E. Ontell M. Cox R. Sassoon D. Buckingham M. (1990). The expression of myosin genes in developing skeletal muscle in the mouse embryo. J. Cell Biol. 111, 1465–1476 10.1083/jcb.111.4.1465 2211821 PMC2116224

[b28] Marx F. P. Soehn A. S. Berg D. Melle C. Schiesling C. Lang M. Kautzmann S. Strauss K. M. Franck T. Engelender S. (2007). The proteasomal subunit S6 ATPase is a novel synphilin-1 interacting protein—implications for Parkinson's disease. FASEB J. 21, 1759–1767 10.1096/fj.06-6734com 17327361

[b29] Masiero E. Agatea L. Mammucari C. Blaauw B. Loro E. Komatsu M. Metzger D. Reggiani C. Schiaffino S. Sandri M. (2009). Autophagy is required to maintain muscle mass. Cell Metab. 10, 507–515 10.1016/j.cmet.2009.10.008 19945408

[b30] McPherron A. C. Lawler A. M. Lee S. J. (1999). Regulation of anterior/posterior patterning of the axial skeleton by growth/differentiation factor 11. Nat. Genet. 22, 260–264 10.1038/10320 10391213

[b31] Miniou P. Tiziano D. Frugier T. Roblot N. Le Meur M. Melki J. (1999). Gene targeting restricted to mouse striated muscle lineage. Nucleic Acids Res. 27, e27–e30 10.1093/nar/27.19.e27 10481039 PMC148637

[b32] Mizushima N. Yoshimori T. Levine B. (2010). Methods in mammalian autophagy research. Cell 140, 313–326 10.1016/j.cell.2010.01.028 20144757 PMC2852113

[b33] Mourkioti F. Slonimsky E. Huth M. Berno V. Rosenthal N. (2008). Analysis of CRE-mediated recombination driven by myosin light chain 1/3 regulatory elements in embryonic and adult skeletal muscle: a tool to study fiber specification. Genesis 46, 424–430 10.1002/dvg.20419 18693277

[b34] Nishino I. Fu J. Tanji K. Yamada T. Shimojo S. Koori T. Mora M. Riggs J. E. Oh S. J. Koga Y. (2000). Primary LAMP-2 deficiency causes X-linked vacuolar cardiomyopathy and myopathy (Danon disease). Nature 406, 906–910 10.1038/35022604 10972294

[b35] Nogalska A. Terracciano C. D'Agostino C. King Engel W. Askanas V. (2009). p62/SQSTM1 is overexpressed and prominently accumulated in inclusions of sporadic inclusion-body myositis muscle fibers, and can help differentiating it from polymyositis and dermatomyositis. Acta Neuropathol. 118, 407–413 10.1007/s00401-009-0564-6 19557423

[b36] Raben N. Hill V. Shea L. Takikita S. Baum R. Mizushima N. Ralston E. Plotz P. (2008). Suppression of autophagy in skeletal muscle uncovers the accumulation of ubiquitinated proteins and their potential role in muscle damage in Pompe disease. Hum. Mol. Genet. 17, 3897–3908 10.1093/hmg/ddn292 18782848 PMC2638578

[b37] Ramachandran N. Munteanu I. Wang P. Ruggieri A. Rilstone J. J. Israelian N. Naranian T. Paroutis P. Guo R. Ren Z. P. (2013). VMA21 deficiency prevents vacuolar ATPase assembly and causes autophagic vacuolar myopathy. Acta Neuropathol. 125, 439–457 10.1007/s00401-012-1073-6 23315026

[b38] Sakao Y. Kawai T. Takeuchi O. Copeland N. G. Gilbert D. J. Jenkins N. A. Takeda K. Akira S. (2000). Mouse proteasomal ATPases Psmc3 and Psmc4: genomic organization and gene targeting. Genomics 67, 1–7 10.1006/geno.2000.6231 10945464

[b39] Salajegheh M. Pinkus J. L. Taylor J. P. Amato A. A. Nazareno R. Baloh R. H. Greenberg S. A. (2009). Sarcoplasmic redistribution of nuclear TDP-43 in inclusion body myositis. Muscle Nerve 40, 19–31 10.1002/mus.21386 19533646 PMC2700211

[b40] Salminen A. Kaarniranta K. (2009). Regulation of the aging process by autophagy. Trends Mol. Med. 15, 217–224 10.1016/j.molmed.2009.03.004 19380253

[b41] Sandri M. Sandri C. Gilbert A. Skurk C. Calabria E. Picard A. Walsh K. Schiaffino S. Lecker S. H. Goldberg A. L. (2004). Foxo transcription factors induce the atrophy-related ubiquitin ligase atrogin-1 and cause skeletal muscle atrophy. Cell 117, 399–412 10.1016/S0092-8674(04)00400-3 15109499 PMC3619734

[b42] Stitt T. N. Drujan D. Clarke B. A. Panaro F. Timofeyva Y. Kline W. O. Gonzalez M. Yancopoulos G. D. Glass D. J. (2004). The IGF-1/PI3K/Akt pathway prevents expression of muscle atrophy-induced ubiquitin ligases by inhibiting FOXO transcription factors. Mol. Cell 14, 395–403 10.1016/S1097-2765(04)00211-4 15125842

[b43] Suzuki N. Motohashi N. Uezumi A. Fukada S. Yoshimura T. Itoyama Y. Aoki M. Miyagoe-Suzuki Y. Takeda S. (2007). NO production results in suspension-induced muscle atrophy through dislocation of neuronal NOS. J. Clin. Invest. 117, 2468–2476 10.1172/JCI30654 17786240 PMC1952622

[b44] Tashiro Y. Urushitani M. Inoue H. Koike M. Uchiyama Y. Komatsu M. Tanaka K. Yamazaki M. Abe M. Misawa H. (2012). Motor neuron-specific disruption of proteasomes, but not autophagy, replicates amyotrophic lateral sclerosis. J. Biol. Chem. 287, 42984–42994 10.1074/jbc.M112.417600 23095749 PMC3522293

[b45] Todde V. Veenhuis M. van der Klei I. J. (2009). Autophagy: principles and significance in health and disease. Biochim. Biophys. Acta 1792, 3–13 10.1016/j.bbadis.2008.10.016 19022377

[b46] Tresse E. Salomons F. A. Vesa J. Bott L. C. Kimonis V. Yao T. P. Dantuma N. P. Taylor J. P. (2010). VCP/p97 is essential for maturation of ubiquitin-containing autophagosomes and this function is impaired by mutations that cause IBMPFD. Autophagy 6, 217–227 10.4161/auto.6.2.11014 20104022 PMC2929010

[b47] van Eersel J. Ke Y. D. Gladbach A. Bi M. Götz J. Kril J. J. Ittner L. M. (2011). Cytoplasmic accumulation and aggregation of TDP-43 upon proteasome inhibition in cultured neurons. PLoS ONE 6, e22850 10.1371/journal.pone.0022850 21829535 PMC3146516

[b48] Vellas B. Pahor M. Manini T. Rooks D. Guralnik J. M. Morley J. Studenski S. Evans W. Asbrand C. Fariello R. (2013). Designing pharmaceutical trials for sarcopenia in frail older adults: EU/US Task Force recommendations. J. Nutr. Health Aging 17, 612–618 10.1007/s12603-013-0362-7 23933872 PMC4077187

[b49] Watts G. D. Wymer J. Kovach M. J. Mehta S. G. Mumm S. Darvish D. Pestronk A. Whyte M. P. Kimonis V. E. (2004). Inclusion body myopathy associated with Paget disease of bone and frontotemporal dementia is caused by mutant valosin-containing protein. Nat. Genet. 36, 377–381 10.1038/ng1332 15034582

[b50] Weihl C. C. Pestronk A. (2010). Sporadic inclusion body myositis: possible pathogenesis inferred from biomarkers. Curr. Opin. Neurol. 23, 482–488 10.1097/WCO.0b013e32833d3897 20664349 PMC3606555

[b51] Winder S. J. Lipscomb L. Angela Parkin C. Juusola M. (2011). The proteasomal inhibitor MG132 prevents muscular dystrophy in zebrafish. PLoS Curr. 3, RRN1286 10.1371/currents.RRN1286 22130468 PMC3219425

[b52] Yamashita S. Kimura E. Tawara N. Sakaguchi H. Nakama T. Maeda Y. Hirano T. Uchino M. Ando Y. (2013). Optineurin is potentially associated with TDP-43 and involved in the pathogenesis of inclusion body myositis. Neuropathol. Appl. Neurobiol. 39, 406–416. 22860700 10.1111/j.1365-2990.2012.01297.x

[b53] Zhao J. Brault J. J. Schild A. Cao P. Sandri M. Schiaffino S. Lecker S. H. Goldberg A. L. (2007). FoxO3 coordinately activates protein degradation by the autophagic/lysosomal and proteasomal pathways in atrophying muscle cells. Cell Metab. 6, 472–483 10.1016/j.cmet.2007.11.004 18054316

