# Supplementary material for: Proteasome dysfunction induces muscle growth defects and protein aggregation
Source: J Cell Sci. 2014 Dec 15;127(24):5204–17. doi: 10.1242/jcs.150961 (PMC4265737; doi:10.1242/jcs.150961)
Supplement: Supplementary Material [file supp_127_24_5204__index.html]

Proteasome dysfunction induces muscle growth defects and protein aggregation — Supplementary Material 

# Proteasome dysfunction induces muscle growth defects and protein aggregation

## JCS150961 Supplementary Material

**Files in this Data Supplement:**

- **Supplementary Material**
