# Supplementary material for: Proteasome dysfunction induces muscle growth defects and protein aggregation
Source: J Cell Sci. 2014 Dec 15;127(24):5204–17. doi: 10.1242/jcs.150961 (PMC4265737; doi:10.1242/jcs.150961)
Supplement: Supplementary Material [file supp_127.24.5204_JCS150961.pdf]

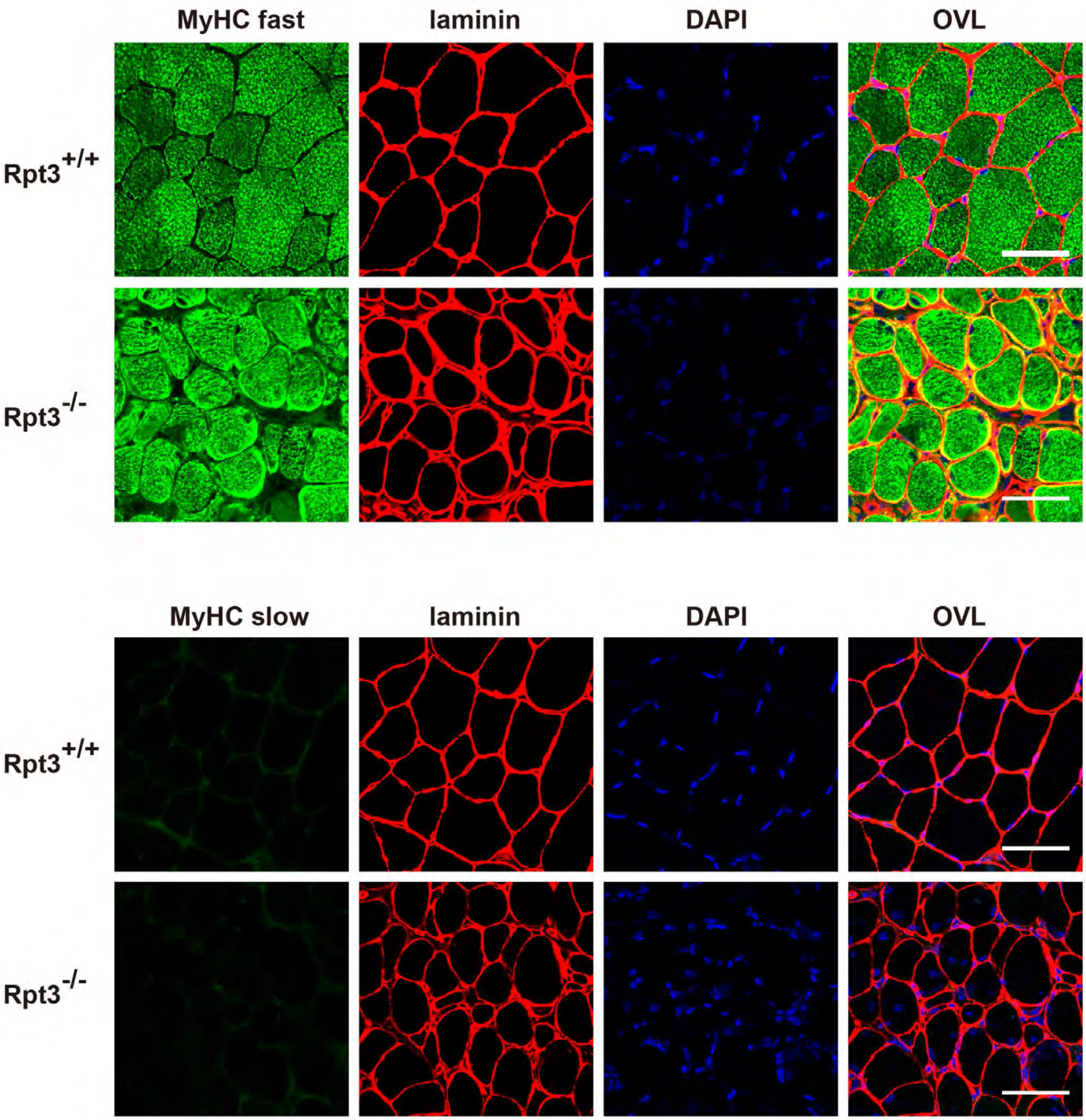

Supplementary Figure 1. Immunohistochemical detection of MyHC fast and MyHC slow in Rpt3$^{+/+}$ and Rpt3$^{-/-}$ mice of the tibialis anterior muscles revealed the muscles contained predominantly MyHC fast fibers. Bar: 50 µm.

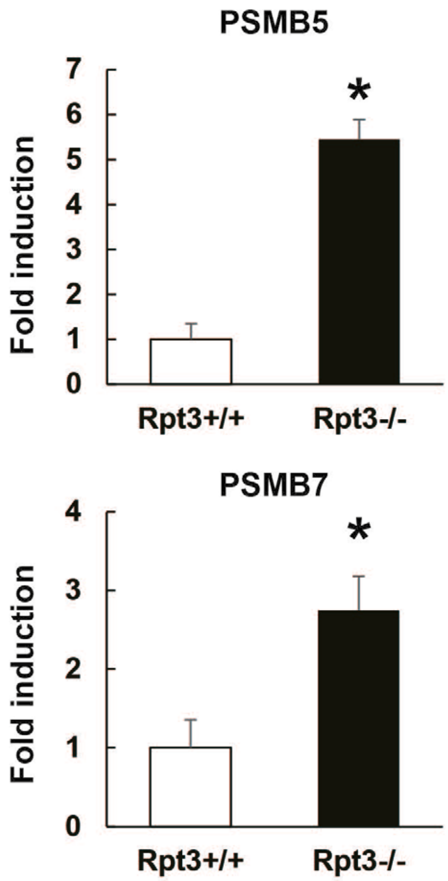

Supplementary Figure 2. The proteolytic subunits in the 20S catalytic core: PSMB5 with chymotrypsin-like activity; and PSMB7 with trypsin-like activity. All of the constitutive proteolytic subunits PSMB5, 7 showed increase expression at the mRNA level in $Rpt3^{-/-}$ mice at the age of 6 weeks. RNA was extracted from the tibialis anterior muscles, and quantitative PCR analysis was performed in triplicates using specific primers (Supplementary Table 1). Data were normalized to the GAPDH content and expressed as fold increase over levels of $Rpt3^{+/+}$ mice. Student's t-test, * $p<0.05$ (n=5).

Supplementary Table 1. Primers for quantitative PCR used in Figure 5B, 5E, 6B and Supplementary Figure 2.

UCHL1-F376	CCCGAAGATAGAGCCAAGTG	UCHL1-R530	CCATCGAGCTCGTACAGATG
Gdf15-F643	CTTGAAGACTTGGGCTGGAG	Gdf15-R838	TAAGAACCACCGGGGTGTAG
CathepsinS-F509	TCAGTGCTCAGAACCTGGTG	CathepsinS-R664	ACTTTTCATCCGTGGCTTTG
FBXO32(Atrogin-1)-F531	ATGCACACTGGTGCAGAGAG	FBXO32(Atrogin-1)-R698	TGTAAGCACACAGGCAGGTC
Fbxo2-F522	CTCCTCCTTCGAGTGGTGTC	Fbxo2-R693	CCTGTCCTGTAGCGAACTCC
PSMB9-F412	ATGCTAATTCGACAGCCCTTTA	PSMB9-R596	GTGATGGTGACCAGGTAGATGA
Pomp-F90	TCTCCGGAAAGGGTTTTCTT	Pomp-R326	TCGTTGCCCCTCAAAATATC
PSME2-F305	CCTTGCTCGCTTTGGTTAAG	PSME2-R540	CTTGGAAGCCTTGGCTACAG
PSMB5-F160	ATCGAAATGCTTCACGGAAC	PSMB5-R395	CGTTCCTTATTGCGAAGCTC
Fgf9-F364	GGGGAGCTGTATGGATCAGA	Fgf9-R589	CTTTGTCAGGGTCCACTGGT
Sdc4-F262	AACCACATCCCTGAGAATGC	Sdc4-R491	AGGAAAACGGCAAAGAGGAT
PSMB4-F260	TTATGCGAGTCAACGACAGC	PSMB4-R494	CTTTCTCCGTCAGCATAGCC
PSMD4-F595	CTTGGTGCCAGTGACTTTGA	PSMD4-R749	GTAGCAATTCCAGCCTCAGC
PSMD5-F306	CACTCTGTCGCAGATTGGAA	PSMD5-R474	GCCAGCCTGGGTTAGTGATA
Vcpip1-F2344	GATGGACGGCAGTCTATGGT	Vcpip1-R2580	AGTGGATGGACCACCTTCTG
PSMB2-F314	CTGGCTATGACGAGCATGAA	PSMB2-R503	TGGAGCTCCTCCAGACACTT
PSMD3-F782	ACCAGGCTGAGAAGCTGGTA	PSMD3-R954	AACAGCTGTGTGCTGTGGAG
PSMD11-F343	GAATGGGCCAAATCAGAGAA	PSMD11-R502	CCACCAAAAGGGCTTTATCA
PSMA2-F342	AGCCTCTGTGATGCAGGAGT	PSMA2-R520	GGAAAGTTTTCCCGTTCACA
PSMD13-F815	TCCAGCTGTTGTGCCTTATG	PSMD13-R1052	TCCTTCATCCCCTTGATCTG
PSMB6-F193	GACAAGCTGACCCCTATCCA	PSMB6-R397	TGATGATTCCTGCCATCAGA
PSMC5-F391	GTGGACCCTTTGGTGTCACT	PSMC5-R599	GCCAACAATGTCTTCCCAGT
PSMC4-F463	ACCTCAGACCAGAAGCCAGA	PSMC4-R693	CACCACACGGATAAATGCAG
Mstn-F716	CTGTAACCTTCCCAGGACCA	Mstn-R912	TCTTTTGGGTGCGATAATCC
Mbnl1-F181	AAAGGTCGTTGCTCCAGAGA	Mbnl1-R378	GCTTGGTGCAACTGAAAACA
MuRF1-F	ACCTGCTGGTGGAAAACATC	MuRF1-R	CTTCGTGTTCCTTGCACATC
p62-F	AGAATGTGGGGAGAGTGTG	p62-R	TCGTCTCCTCCTGAGCAGTT
GAPDH-F	AACTTTGGCATTGTGGAAGG	GAPDH-R	CACATTGGGGGTAGGAACAC
PSMB7-F	GTGTCGGTGTTTCAGCCAC	PSMB7-R	TCCGCTTCCAAGACAGCATTC

Supplementary Table 2. Top 10 networks identified by ingenuity pathway analysis.

	Associated Network Functions	Score
1	Connective Tissue Disorders, Genetic Disorder, Dermatological Diseases and Conditions	60
	Major interactors: TGFbeta1, collagen	
2	Cellular Development, Embryonic Development, Tissue Development	56
3	Protein Synthesis, Molecular Transport, Protein Trafficking	54
	Major interactors:60S ribosomal subunit, MAP3K14	
4	Carbohydrate Metabolism, Small Molecule Biochemistry, Nucleic Acid Metabolism	54
5	Cell Cycle, Cellular Compromise, Cell Death	52
6	Nucleic Acid Metabolism, Small Molecule Biochemistry, Developmental Disorder	52
7	Developmental Disorder, Genetic Disorder, Metabolic Disease	51
8	Carbohydrate Metabolism, Small Molecule Biochemistry, Cell Signaling	50
9	Genetic Disorder, Metabolic Disease, Cardiovascular Disease	48
10	Cell Death, DNA Replication, Recombination, and Repair, Cell Morphology	46

Supplementary Table 3. KEGG Pathway analysis using the differentially expressed genes.

KEGG_PATHWAY analysis using the differentially expressed genes in comparison of Rpt3+/+ with −/− mice

Term	Count	PValue	Fold Enrichment	Bonferroni	Benjamini	FDR
mmu03050:Proteasome	25	1.76E−08	3.368794326	3.28E−06	3.28E−06	2.19E−05
mmu03010:Ribosome	36	5.71E−08	2.561797753	1.06E−05	5.31E−06	7.07E−05
mmu04142:Lysosome	34	5.63E−04	1.80952381	0.099514555	0.034337057	0.695527923
mmu04510:Focal adhesion	48	0.001926039	1.535353535	0.301338299	0.085746284	2.359401667
mmu05410:Hypertrophic cardiomyopathy (HCM)	25	0.001983154	1.884920635	0.30873557	0.071185809	2.428580024
mmu04810:Regulation of actin cytoskeleton	50	0.004601206	1.459293395	0.57590561	0.133216928	5.551645387
mmu00280:Valine, leucine and isoleucine degradation	15	0.009119711	2.065217391	0.818054551	0.216069947	10.72645197
mmu04530:Tight junction	33	0.009772539	1.548148148	0.839045305	0.204137657	11.45216538
mmu04920:Adipocytokine signaling pathway	19	0.013535507	1.7960199	0.920722286	0.245457933	15.53060216
mmu05414:Dilated cardiomyopathy	24	0.014045381	1.652173913	0.927990756	0.231331959	16.06973474
mmu04666:Fc gamma R−mediated phagocytosis	25	0.015808901	1.615646259	0.948385163	0.236200261	17.91015851
mmu05416:Viral myocarditis	24	0.018093978	1.617021277	0.966503013	0.246499037	20.23970867
mmu00604:Glycosphingolipid biosynthesis	7	0.021105157	2.955555556	0.981080768	0.263022691	23.21671044
mmu05210:Colorectal cancer	22	0.023742285	1.620155039	0.988545052	0.273297506	25.73964253
mmu00480:Glutathione metabolism	15	0.027081582	1.826923077	0.993943593	0.288544619	28.82497616
mmu00511:Other glycan degradation	7	0.029370817	2.770833333	0.996092292	0.292876722	30.87154199
mmu04512:ECM−receptor interaction	21	0.030745987	1.602409639	0.996998108	0.289422467	32.07478509
mmu04010:MAPK signaling pathway	54	0.035198094	1.290566038	0.99872504	0.309451408	35.8394682
mmu05214:Glioma	17	0.036692318	1.682291667	0.99904436	0.306465414	37.05933728
mmu04910:Insulin signaling pathway	31	0.037693063	1.422705314	0.999212351	0.300453429	37.86437415
mmu04662:B cell receptor signaling pathway	20	0.039696027	1.583333333	0.999465407	0.301459536	39.44727586
mmu00240:Pyrimidine metabolism	23	0.040983177	1.517361111	0.99958344	0.297980786	40.44482842
mmu05218:Melanoma	18	0.046575138	1.605633803	0.999859627	0.320028668	44.60572661
mmu00230:Purine metabolism	34	0.046647958	1.371549894	0.999861607	0.309421453	44.6581025
mmu04062:Chemokine signaling pathway	38	0.056979307	1.322344322	0.999981763	0.353694435	51.6442983
mmu05200:Pathways in cancer	62	0.068921457	1.215686275	0.999998296	0.400025363	58.70491074
mmu05412:Arrhythmogenic right ventricular cardiomyopathy (ARVC)	18	0.072807988	1.52	0.999999217	0.405932789	60.78975733
mmu04664:Fc epsilon RI signaling pathway	19	0.085617609	1.467479675	0.999999941	0.44820484	66.99558386
mmu00760:Nicotinate and nicotinamide metabolism	8	0.086108664	2.026666667	0.999999947	0.438711944	67.21443035
mmu04144:Endocytosis	40	0.098097669	1.254125413	0.999999995	0.472783541	72.16093405
